# Hospital-Based Rehabilitation, Functional Ambulation Status at Discharge, and Hospital Outcomes Across Society of Thoracic Surgeons Risk Groups in Adult Patients After Transcatheter Aortic Valve Replacements

**DOI:** 10.7759/cureus.102918

**Published:** 2026-02-03

**Authors:** Adele L Myszenski, Karen Childers, Kelsey Seifferlein, Alison Wickenheiser, Jessica Gibson, Ryann Laier, George Divine

**Affiliations:** 1 Rehabilitation, Henry Ford Health System, Detroit, USA; 2 Physiology, Michigan State University College of Human Medicine, East Lansing, USA; 3 Physical Therapy, Wayne State University, Detroit, USA; 4 Public Health Sciences, Henry Ford Health System, Detroit, USA

**Keywords:** ambulation status, discharge disposition, functional outcomes, hospital-based rehabilitation, length of stay, occupational therapy, physical therapy, society of thoracic surgeons risk score, transcatheter aortic valve replacement

## Abstract

Introduction

Hospital-based rehabilitation after transcatheter aortic valve replacement (TAVR) is novel in the literature. This study aimed to examine clinical outcomes across Society of Thoracic Surgeons (STS) risk scores for patients after TAVR.

Methods

The study population included 1321 consecutive TAVR recipients. Patients were divided into three groups: low (<4), intermediate (4.0-7.9), and high (>8) STS risk score. Outcomes included receipt of hospital-based physical therapy (PT) or occupational therapy (OT), hospital length of stay (LOS), home vs non-home disposition, and functional ambulation status at discharge.

Results

A total of 821 patients (62%) received PT or OT visits; patients in the high and intermediate risk groups had 3.85 and 2.19 times higher odds of receiving PT or OT, respectively. LOS was significantly higher in high and intermediate risk groups (1.99 and 1.48 times longer, respectively; p<0.001). The odds of discharge to home were 61% lower for the intermediate compared to the low group and 79% lower for the high compared to the low group (p<0.001). The odds of being functionally ambulatory at discharge were 50% lower between intermediate and low groups and 70% lower between high and low groups.

Conclusions

TAVR recipients who had high STS risk scores were more likely to receive PT or OT and have longer LOS, and less likely to be functionally ambulatory or to return home at discharge. A clinical pathway based on STS risk level could help with patient selection for hospital-based PT or OT.

## Introduction

Structural heart procedures, including transcatheter aortic valve replacement (TAVR), have emerged as an effective alternative medical intervention to traditional open-heart procedures for patients with symptomatic aortic valve disease [1]. In 2012, TAVR procedures were approved by the US Food and Drug Administration for patients with high to prohibitive risk for open-heart surgery via sternotomy and standard aortic valve replacement (AVR). The US Food and Drug Administration (FDA) approved the procedure for patients with intermediate risk in 2016 and for low-risk in 2019 [2-4]. Risk is scored based on the Society of Thoracic Surgeons (STS) risk categorization [5]. STS risk categories originally described risk related to coronary artery bypass graft procedures but have since expanded to describe risk in other procedures, including TAVR [6]. Typically, patients with higher STS scores are older and have more clinical and anatomic risk factors [7].

Following a TAVR, patients may also have functional impairments and lower quality of life scores based on the Euro-Quality of Life, Kansas City Cardiomyopathy Questionnaire, or Minnesota Living with Heart Failure Questionnaire [8,9]. Hospitalized patients with functional disabilities or impairments are more likely to have lower mobility scores, longer lengths of stay (LOS), and higher rates of discharge to post-acute rehabilitation facilities, all of which are correlated with higher health care costs in the literature [10,11]. Studies have shown that timing and frequency of physical therapy (PT) and occupational therapy (OT) services could impact functional ability, LOS, and discharge rates to skilled nursing facilities [12-14]. Intervention is individualized for the patient's specific basic activities of daily living, instrumental activities of daily living, and mobility impairments and titrated based on physiological response. A prior single-center study of patients with high-risk STS scores found that the implementation of PT and OT structural heart pathway following TAVR improved LOS and discharge to home for those without major events [15]. There is a lack of studies that include patients in low- or intermediate-risk categories or those with post-procedural events. 

The primary aim of this study was to examine the relationship between the STS risk score and hospital-based PT or OT receipt, hospital LOS, and discharge rates to home versus not home for patients post-TAVR procedure. For those who received hospital-based PT or OT, the impact of STS risk score on functional ambulation status at discharge and receipt of PT or OT on the first day after the procedure was analyzed.

## Materials and methods

Study design and population

This was a single-center retrospective cohort study of adults who underwent a TAVR and were admitted after September 1, 2019, and discharged before December 31, 2023, at Henry Ford Hospital, an 877-bed, academic, level I trauma center in Detroit, US. Patients were stratified into three cohorts based on operative STS risk score category at time of pre-clinical evaluation: low (0-3.9), intermediate (4.0-7.9), and high (8.0+) [2,3].

A primary analysis of all patients was completed. The primary outcome variables were receipt of one or more hospital-based PT or OT visits during the hospitalization in which the TAVR procedure took place, hospital LOS, and discharge destination (home or not home). Demographic, preclinical, and intra-procedure data, occurrence of adverse events, and hospital outcomes were compared between groups. A secondary analysis of patients who received at least one PT or OT visit was completed for the same variables, as well as additional rehabilitation and functional outcome variables.

Demographic and clinical outcomes

Demographics were extracted from the electronic health record, including age, sex, race, insurance, and body mass index. Comorbidity burden was quantified by using the Charlson Comorbidity Index and the number of previous cardiac surgeries [16].

Preclinical data collected from the STS/American College of Cardiology Transcatheter Valve Therapy (TVT) registry included a gait speed test, the five-meter walking test, and a quality-of-life outcome measure, the Kansas City Cardiomyopathy Questionnaire-12, a 12-item patient-reported outcome questionnaire that measures the health status in those diagnosed with heart failure. The five-meter walk test is a standardized gait speed assessment performed over three consecutive trials, averaged, and then recorded in seconds. Speeds >6 seconds are defined as slow and frail, and speeds <6 seconds are defined as normal and not-frail [17,18]. The Kansas City Cardiomyopathy Questionnaire-12 consists of four domains: physical limitation, symptom frequency, quality of life, and social limitation. Scales from each domain range from 0 (worst health status) to 100 (best possible health status), which can then be combined into an overall summary score [19]. Other clinical data collected from the TVT registry included the transcatheter access site and occurrence of adverse events (major vs minor). Clinical outcomes collected from the hospital's electronic medical record data warehouse included hospital LOS, days on ventilator, days in intensive care unit, and mortality.

Hospital-based rehabilitation intervention variables

Rehabilitation-specific variables included received PT or OT (yes/no), number of PT visits, number of OT visits, number of combined PT and OT visits (acute rehabilitation visits), and visit on post-procedure day 1 (yes/no). Two prospectively collected functional outcome tools were collected by trained physical therapists, PT assistants, occupational therapists, and OT assistants for every PT and OT visit. The Activity Measure for Post-Acute Care "6 clicks" and the Johns Hopkins Highest Level of Mobility were selected based on strong evidence for reliability, validity, and recording at each PT visit [20-22]. During the study timeframe, only PTs scored the Activity Measure for Post-Acute Care "6 clicks" Basic Mobility tool, and only OTs scored the Activity Measure for Post-Acute Care "6 clicks" Daily Activity tool. Both PT and OT score the Johns Hopkins Highest Level of Mobility at each visit. Scores from the first and last visits were reported. Patients were considered functionally ambulatory at discharge if their score on the last PT or OT visit was a 7 or 8 on the Johns Hopkins Highest Level of Mobility (ambulated more than 25 feet) or higher than a 21 on the "6 clicks" Basic Mobility (supervision or independent for all levels of activity, including gait and stairs). 

Institutional standards for hospital-based rehabilitation

A prior study includes the guidelines for rehabilitation for patients following TAVR at our institution at the time when FDA approval included patients in the high-risk category [15]. While this guideline continues to be followed (since 2014), an additional screening of frailty was developed by a collaborative decision-making group involving cardiologists, advanced practice providers, and therapists and incorporated into the rehabilitation pathway in 2022. Screening was based on a quality-improvement protocol piloted in 2021 as the Next Day Discharge protocol [23]. Preclinical screening by an advanced practice provider included a hospital-specific frailty score of 0-3 points (1 point is scored for patients with a five-meter walk test score >6 seconds; 1 point for a Katz basic activities of daily living score <6, and 1 point if blood albumin <3.5 mg/dL) [24-26]. Scores were provided to the rehab department via an emailed list of patients scheduled for procedures in the upcoming week. Patients with a frailty score of 1 were scheduled to be seen by either PT or OT. Patients scoring 2 or 3 points were assigned to both PT and OT. Any provider was able to consult rehabilitation regardless of the frailty score, and therefore, this study design did not stratify by this score.

In addition to a PT or OT evaluation of function and physiologic tolerance of mobility and activities of daily living, patients who received one or more PT or OT visits were provided with education on energy conservation, expected changes following the procedure, cardiac warning signs, education on a home walking program, and education on stage II cardiac rehabilitation. Additionally, all patients were to receive a cardiac rehab phase II referral by advanced practice providers.

Data extraction

Although the data for this study was analyzed retrospectively, the Activity Measure for Post-Acute Care "6 clicks" Basic Mobility tool, Activity Measure for Post-Acute Care "6 clicks" Daily Activity tool, and the Johns Hopkins Highest Level of Mobility scale were collected prospectively. The initial sample of subjects was received by the authors via a spreadsheet file from the STS/American College of Cardiology TVT Registry. Subjects were then matched to a clinical rehab database of electronic medical record (EPIC, Epic Systems, Verona, US) data for hospitalized individuals by an experienced programmer. The clinical rehabilitation database includes demographic, rehabilitation, and clinical data that were extracted from the electronic medical record by an expert in data mining and the EPIC medical record. Prior to data analysis, all identifying information was removed from the database.

Data analysis

Continuous variables were summarized as medians with interquartile range, and categorical variables were presented as counts and column percentages. Comparisons across STS risk groups (low, intermediate, and high) were conducted using the Kruskal-Wallis rank-sum test for continuous variables. For categorical variables, Pearson's chi-squared test was used when expected cell counts exceeded five; otherwise, Fisher's exact test was applied. Two variables were created to define an adverse event. The first categorized patients as none, minor, or major: patients with at least one major event were classified as major; if no major event occurred but at least one minor event did, they were classified as minor; otherwise, none. This variable assessed the association with STS risk. The second was a binary indicator (1=any adverse event; 0=none). Univariable and multivariable models were used to assess the association between STS risk and outcomes of interest, with all multivariable models adjusted for the occurrence of at least one adverse event. Logistic regression was used for the binary outcomes of discharge disposition (home vs. not home) and receipt of PT among all patients. Additional logistic models examined the associations between STS risk and receipt of PT or OT on postoperative day 1, as well as functional ambulatory status among patients who received therapy. Generalized linear models with a gamma distribution and log link were used to model hospital LOS for all patients, given the skewed distribution of this outcome. All statistical tests were two-sided, with a significance level set at p<0.05. Analyses were conducted using R Statistical Software (version 4.4.0; R Core Team). 

Ethical considerations and consent 

Study design and data collection methods were approved by the hospital's institutional review board (#17435). Informed consent was waived due to the retrospective nature of the study.

## Results

A total of 1325 patients underwent TAVR from September 1, 2019, to December 31, 2023; four patients were excluded because STS risk information was not available.

Clinical characteristics of all patients

Clinical characteristics for all patients are described in Table 1. There were 244 patients with STS risk scores of 8% or higher (high-risk group), 390 patients had scores of 4.1-7.9% (intermediate risk group), and 687 patients had scores of 3.9% or lower (low risk group). Patients having higher STS risk were older, with median ages of 83 years (high), 81 years (intermediate), and 75 years (low). Most patients with low STS risk were male (421/687, 61.3%), while females were more common in the intermediate (213/390, 54.6%) and high risk (162/244, 66.4%) groups. Most patients were White (low: 528/687, 76.9%; intermediate: 294/390, 75.4%; high: 178/244, 73%) and were accessed by femoral artery (low: 624/687, 90.8%; intermediate: 347/390, 89%; high: 195/244, 79.9%). As STS risk increased, patients were more likely to experience an adverse event (low: 145/687, 21.1%, intermediate: 106/390, 27.1%; high: 81/244, 33.2%). Specific event details are reported in Table 2.

**Table 1:** Summary of patient baseline characteristics, clinical and surgical measures, and outcomes by STS risk category for patients who received TAVR ^1^Kruskal-Wallis rank sum test; ^2^Pearson's chi-squared test; ^3^Fisher's exact test with simulated p-value; ^4^Fisher's exact test CCI - Charlson Comorbidity Index; ICU - intensive care unit; KCCQ-12 - Kansas City Cardiomyopathy Questionnaire-12; LOS - length of stay; OT - occupational therapy; PT - physical therapy; STS - Society of Thoracic Surgeons; TVT - transcatheter valve therapy; TAVR - transcatheter aortic valve replacement

Variable; median (IQR), n (%)	n	STS low risk, n=687	STS intermediate risk, n=390	STS high risk, n=244	p-value
Age, years	1321	75.0 (69.0-81.0)	81.0 (75.0-86.8)	83.0 (77.8-89.0)	<0.001^1^
Sex					
Male	1321	421 (61.3%)	177 (45.4%)	82 (33.6%)	<0.001^2^
Female		266 (38.7%)	213 (54.6%)	162 (66.4%)	
Race					
American Indian/Alaska Native	1321	2 (0.3%)	0 (0.0%)	0 (0.0%)	0.41^3^
Asian		7 (1.0%)	3 (0.8%)	6 (2.5%)	
Black		99 (14.4%)	62 (15.9%)	36 (14.8%)	
Hispanic		1 (0.1%)	0 (0.0%)	0 (0.0%)	
Native Hawaiian/Pacific Islander		0 (0.0%)	0 (0.0%)	1 (0.4%)	
White		528 (76.9%)	294 (75.4%)	178 (73.0%)	
Other		13 (1.9%)	7 (1.8%)	2 (0.8%)	
Do not know/declined		37 (5.4%)	24 (6.2%)	21 (8.6%)	
Race - recategorized					
Black	1321	99 (14.4%)	62 (15.9%)	36 (14.8%)	0.61
White		528 (76.9%)	294 (75.4%)	178 (73.0%)	
Do not know/declined		37 (5.4%)	24 (6.2%)	21 (8.6%)	
Other		23 (3.3%)	10 (2.6%)	9 (3.7%)	
Insurance					
Medicaid	1321	13 (1.9%)	5 (1.3%)	0 (0.0%)	<0.001^3^
Medicare		567 (82.5%)	354 (90.8%)	235 (96.3%)	
Private		107 (15.6%)	31 (7.9%)	9 (3.7%)	
Body mass index, kg/m^2^	1316	29.1 (25.4-33.6)	28.1 (24.0-32.1)	26.7 (22.9-31.8)	<0.001^1^
CCI	1321	2.0 (1.0-4.0)	4.0 (2.0-6.0)	5.0 (3.0-7.0)	<0.001^1^
Overall KCCQ-12	1297	59.9 (38.5-80.2)	43.8 (25.1-65.6)	34.4 (19.8-54.7)	<0.001^1^
Number of previous cardiac surgeries	1311	0.0 (0.0-0.0)	0.0 (0.0-1.0)	0.0 (0.0-1.0)	<0.001^1^
TVT access site					
Axillary artery	1321	7 (1.0%)	5 (1.3%)	6 (2.5%)	0.004^3^
Direct aortic		1 (0.1%)	0 (0.0%)	0 (0.0%)	
Femoral artery		624 (90.8%)	347 (89.0%)	195 (79.9%)	
Subclavian artery		2 (0.3%)	6 (1.5%)	4 (1.6%)	
Transapical		1 (0.1%)	0 (0.0%)	0 (0.0%)	
Transcarotid		11 (1.6%)	7 (1.8%)	6 (2.5%)	
Transcaval		40 (5.8%)	23 (5.9%)	32 (13.1%)	
Transseptal via femoral vein		1 (0.1%)	2 (0.5%)	1 (0.4%)	
TVT access site recategorized					
Femoral artery	1321	624 (90.8%)	347 (89.0%)	195 (79.9%)	0.001^3^
Subclavian artery		2 (0.3%)	6 (1.5%)	4 (1.6%)	
Other		61 (8.9%)	37 (9.5%)	45 (18.4%)	
TVT access site: femoral	1321	624 (90.8%)	347 (89.0%)	195 (79.9%)	<0.001^2^
Received PT or OT	1321	351 (51.1%)	273 (70.0%)	197 (80.7%)	<0.001^2^
Days on ventilator	55	4.0 (2.0-11.0)	3.0 (2.0-5.0)	2.0 (2.0-4.0)	0.39^1^
Days in ICU	149	2.0 (1.0-5.0)	3.0 (2.0-6.0)	3.0 (2.0-7.0)	0.39^1^
Expired during hospital stay	1321	5 (0.7%)	8 (2.1%)	6 (2.5%)	0.05^4^
Discharge destination: home	1321	655 (95.3%)	344 (88.2%)	194 (79.5%)	<0.001^2^
Hospital LOS (days)	1321	1.0 (1.0, 2.0)	1.0 (1.0, 3.0)	2.0 (1.0, 6.0)	<0.001^1^
Adverse event					
No adverse event	1321	542 (78.9%)	284 (72.8%)	163 (66.8%)	0.004^2^
Minor adverse event		21 (3.1%)	15 (3.8%)	13 (5.3%)	
Major adverse event		124 (18.0%)	91 (23.3%)	68 (27.9%)	

**Table 2:** Summary of adverse events by STS risk category for patients who received TAVR ^1^Minor event post procedure; ^2^Major event post procedure ASD - atrial septal defect; ICD - implanted cardiac device; STS - Society of Thoracic Surgeons; TAVR - transcatheter aortic valve replacement

Post-procedure adverse event; n (%)	Overall, n=1321	STS low risk, n=687	STS intermediate risk, n=390	STS high risk, n=244
ASD closure^1^	0 (0.0%)	0 (0.0%)	0 (0.0%)	0 (0.0%)
Coronary compression or obstruction^1^	2 (0.2%)	2 (0.3%)	0 (0.0%)	0 (0.0%)
COVID-positive^1^	3 (0.2%)	0 (0.0%)	1 (0.3%)	2 (0.8%)
Device embolization^1^	6 (0.5%)	2 (0.3%)	2 (0.5%)	2 (0.8%)
Device migration^1^	5 (0.4%)	2 (0.3%)	1 (0.3%)	2 (0.8%)
Device recapture or retrieval^1^	4 (0.3%)	1 (0.1%)	2 (0.5%)	1 (0.4%)
Device-related event: other^1^	4 (0.3%)	3 (0.4%)	1 (0.3%)	0 (0.0%)
Percutaneous coronary intervention^1^	57 (4.3%)	22 (3.2%)	14 (3.6%)	21 (8.6%)
Vascular complication: minor^1^	52 (3.9%)	21 (3.1%)	16 (4.1%)	15 (6.1%)
Annular rupture^2^	2 (0.2%)	1 (0.1%)	0 (0.0%)	1 (0.4%)
Aortic dissection^2^	3 (0.2%)	0 (0.0%)	2 (0.5%)	1 (0.4%)
Atrial fibrillation^2^	32 (2.4%)	23 (3.3%)	6 (1.5%)	3 (1.2%)
Bleeding: access site^2^	14 (1.1%)	5 (0.7%)	5 (1.3%)	4 (1.6%)
Bleeding: genitourinary^2^	3 (0.2%)	1 (0.1%)	1 (0.3%)	1 (0.4%)
Bleeding: hematoma at access site^2^	29 (2.2%)	14 (2.0%)	11 (2.8%)	4 (1.6%)
Bleeding: other^2^	54 (4.1%)	17 (2.5%)	17 (4.4%)	20 (8.2%)
Bleeding: retroperitoneal^2^	9 (0.7%)	4 (0.6%)	4 (1.0%)	1 (0.4%)
Cardiac arrest^2^	39 (3.0%)	12 (1.7%)	15 (3.8%)	12 (4.9%)
Cardiac perforation^2^	2 (0.2%)	0 (0.0%)	1 (0.3%)	1 (0.4%)
Cardiac surgery or intervention unplanned^2^	25 (1.9%)	12 (1.7%)	7 (1.8%)	6 (2.5%)
Conduction native pacer disturbance required ICD^2^	10 (0.8%)	2 (0.3%)	6 (1.5%)	2 (0.8%)
Conduction native pacer disturbance required pacer^2^	104 (7.9%)	50 (7.3%)	34 (8.7%)	20 (8.2%)
Dialysis new requirement^2^	4 (0.3%)	0 (0.0%)	2 (0.5%)	2 (0.8%)
Mitral valve reintervention^2^	0 (0.0%)	0 (0.0%)	0 (0.0%)	0 (0.0%)
Myocardial infarction^2^	2 (0.2%)	0 (0.0%)	2 (0.5%)	0 (0.0%)
Reintervention aortic valve^2^	2 (0.2%)	0 (0.0%)	1 (0.3%)	1 (0.4%)
Stroke hemorrhagic^2^	3 (0.2%)	0 (0.0%)	2 (0.5%)	1 (0.4%)
Transient ischemic attack^2^	1 (0.1%)	1 (0.1%)	0 (0.0%)	0 (0.0%)
Unplanned vascular surgery or intervention^2^	58 (4.4%)	20 (2.9%)	22 (5.6%)	16 (6.6%)
Vascular complication: major^2^	7 (0.5%)	1 (0.1%)	4 (1.0%)	2 (0.8%)

All patients: LOS by STS

**Figure 1:**
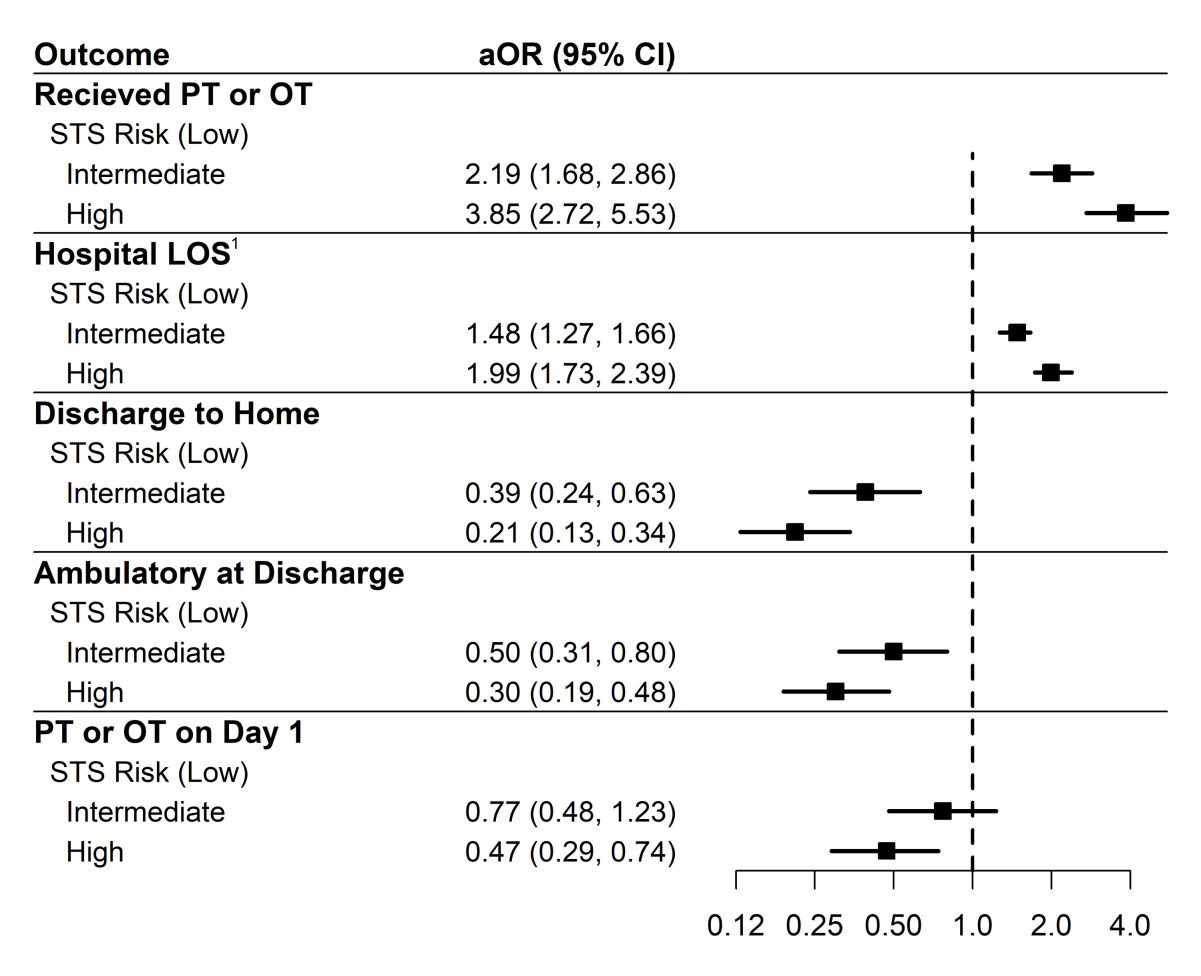
Forest plot showing adjusted odds ratios (aORs) with 95% CIs from logistic regression models for receipt of PT or OT, LOS, discharge to home, ambulatory status at discharge, and receipt of PT or OT on postoperative day 1 ^1^Adjusted exponentiated β coefficients with 95% CIs from a gamma regression model with log link are shown for hospital LOS. All models were adjusted for the presence of an adverse event. STS - Society of Thoracic Surgeons; LOS - length of stay; PT - physical therapy; OT - occupational therapy

Across all patients, there was a statistically significant association between hospital LOS and STS risk group (p<0.001; see Table 1). Patients in the intermediate risk group had a LOS 1.58 times longer than the low STS risk group (exp[β]=1.58; 95% CI:1.32-1.90; p<0.001). Those patients in the high STS risk group had 2.21 times longer LOS than the low risk group (exp[β]=2.21; 95% CI: 1.78-2.75; p<0.001). Results were similar when adjusted for the occurrence of at least one adverse event, with hospital LOS being 1.48 times and 1.99 times longer in the intermediate and high risk groups (Table 3 and Figure 1).

**Table 3:** Model results assessing the association between STS risk group and clinical outcomes ^1^Hospital length of stay was modeled using univariable gamma regression with log link; values reported represent the ratio of expected length of stay relative to the reference group. All other outcomes were modeled using univariable logistic regression; values reported are odds ratios. ^2^Same model, but adjusted for the presence of at least one adverse event. aOR - adjusted odds ratio; OR - odds ratio; OT - occupational therapy; PT - physical therapy; STS - Society of Thoracic Surgeons

Outcome	STS risk	OR (95% CI)^1^	p-value	aOR (95% CI)^2^	p-value
Hospital length of stay	Low	Reference	-	Reference	-
	Intermediate	1.58 (1.32-1.91)	<0.001	1.48 (1.27-1.73)	<0.001
	High	2.21 (1.78-2.75)	<0.001	1.99 (1.66-2.39)	<0.001
Discharge to home (home vs. not home)	Low	Reference	-	Reference	-
	Intermediate	0.37 (0.23-0.58)	<0.001	0.39 (0.24-0.63)	<0.001
	High	0.19 (0.12-0.30)	<0.001	0.21 (0.13-0.34)	<0.001
Receipt of therapy (yes vs. no)	Low	Reference	-	Reference	-
	Intermediate	2.23 (1.72-2.91)	<0.001	2.19 (1.68-2.86)	<0.001
	High	4.01 (2.85-5.76)	<0.001	3.85 (2.72-5.53)	<0.001
PT/OT on day 1 post-procedure (yes vs. no)	Low	Reference	-	Reference	-
	Intermediate	0.72 (0.46-1.13)	0.156	0.77 (0.48-1.23)	0.27
	High	0.42 (0.27-0.65)	<0.001	0.47 (0.29-0.74)	0.001
Functional ambulatory status (yes vs. no)	Low	Reference	-	Reference	-
	Intermediate	0.50 (0.31-0.79)	0.004	0.50 (0.31-0.80)	0.004
	High	0.29 (0.18-0.46)	<0.001	0.30 (0.19-0.48)	<0.001

All patients: discharge home by STS

Across all patients, there was a statistically significant association between discharge destination of home and STS risk group (p<0.001; see Table 1). Patients in the intermediate risk group had 63% lower odds of discharge home than those in the low risk group (OR: 0.37; 95% CI: 0.23-0.58; p<0.001). Those patients in the high STS risk group had 81% lower odds of discharge home than those in the low STS risk group (OR: 0.19; 95% CI: 0.12-0.30; p<0.001). Results were found to be similar when adjusted for the occurrence of at least one adverse event, 61% lower odds and 79% lower odds (Table 3 and Figure 1).

All patients: receipt of PT or OT

Of the total 1321 patients included, 821 (62.2%) received one or more PT or OT visits (see Table 1). The proportion of patients who received PT or OT services increased as the STS risk group increased (351/687, 51.1% for low, 273/390, 70% for intermediate, and 197/244, 80.7% for high groups, p<0.001). Patients in the intermediate STS risk group had 2.23 times higher odds of receiving a visit from at least one acute rehabilitation therapy service than those in the low risk group (OR: 2.23; 95% CI: 1.72-2.91, p<0.001). Those patients in the high STS risk group had 4.01 times higher odds of receiving PT, OT, or both than the low STS risk group (OR: 4.01; 95% CI: 2.85-5.76, p<0.001). Results were similar when adjusted for the occurrence of at least one adverse event, with 2.19 times higher odds and 3.85 times higher odds (Table 3 and Figure 1).

Clinical characteristics of patients who did not receive PT or OT

Five hundred patients (37.9%) did not receive PT or OT services, with the majority being from the low STS risk category (336/500, 67.2%). Between-group differences were statistically significant for demographics, comorbidities, Kansas City Cardiomyopathy Questionnaire-12 scores, and hospital LOS by STS risk group. There were no significant differences found in TVT access site, discharge to home, or in-hospital adverse events (Table 4).

**Table 4:** Summary of patient baseline characteristics, clinical and surgical measures, and outcomes by STS risk category for patients who did not receive any therapy ^1^Kruskal-Wallis rank sum test; ^2^Pearson's chi-squared test; ^3^Fisher's exact test with simulated p-value; ^4^Fisher's exact test. CCI - Charlson Comorbidity Index; ICU - intensive care unit; KCCQ-12 - Kansas City Cardiomyopathy Questionnaire-12; LOS - length of stay; OT - occupational therapy; PT - physical therapy; STS - Society of Thoracic Surgeons; TVT - transcatheter valve therapy

Variable; median (IQR), n (%)	n	STS low risk, n=336	STS intermediate risk, n=117	STS high risk, n=47	p-value
Age, years	500	75.0 (68.0-80.0)	80.0 (75.0-86.0)	84.0 (77.5-87.0)	<0.001^1^
Sex					
Male	500	228 (67.9%)	56 (47.9%)	20 (42.6%)	<0.001^2^
Female		108 (32.1%)	61 (52.1%)	27 (57.4%)	
Race					
American Indian/Alaska Native	500	0 (0.0%)	0 (0.0%)	0 (0.0%)	0.60^3^
Asian		4 (1.2%)	0 (0.0%)	0 (0.0%)	
Black		33 (9.8%)	16 (13.7%)	7 (14.9%)	
Hispanic		1 (0.3%)	0 (0.0%)	0 (0.0%)	
Native Hawaiian/Pacific Islander		0 (0.0%)	0 (0.0%)	0 (0.0%)	
White		273 (81.3%)	90 (76.9%)	35 (74.5%)	
Other		9 (2.7%)	4 (3.4%)	0 (0.0%)	
Do not know/declined		16 (4.8%)	7 (6.0%)	5 (10.6%)	
Insurance					
Medicaid	500	5 (1.5%)	0 (0.0%)	0 (0.0%)	<0.001^3^
Medicare		267 (79.5%)	111 (94.9%)	45 (95.7%)	
Private		64 (19.0%)	6 (5.1%)	2 (4.3%)	
Body mass index, kg/m^2^	499	28.6 (25.6-33.5)	28.2 (23.7-32.5)	25.2 (22.1-30.8)	0.009^1^
CCI	500	2.0 (1.0-4.0)	3.0 (2.0-5.0)	5.0 (3.0-6.0)	<0.001^1^
Overall KCCQ-12	496	69.8 (48.6-83.7)	47.4 (31.3-72.0)	45.8 (31.5-63.3)	<0.001^1^
Number of previous cardiac surgeries	498	0.0 (0.0-0.0)	0.0 (0.0-1.0)	0.0 (0.0-1.0)	<0.001^1^
TVT access site					
Axillary artery	500	4 (1.2%)	0 (0.0%)	0 (0.0%)	0.33^3^
Direct aortic		1 (0.3%)	0 (0.0%)	0 (0.0%)	
Femoral artery		316 (94.0%)	110 (94.0%)	41 (87.2%)	
Subclavian artery		1 (0.3%)	1 (0.9%)	1 (2.1%)	
Transapical		0 (0.0%)	0 (0.0%)	0 (0.0%)	
Transcarotid		0 (0.0%)	0 (0.0%)	0 (0.0%)	
Transcaval		13 (3.9%)	6 (5.1%)	5 (10.6%)	
Transseptal via femoral vein		1 (0.3%)	0 (0.0%)	0 (0.0%)	
TVT access site: femoral	500	316 (94.0%)	110 (94.0%)	41 (87.2%)	0.23^4^
Received PT or OT	500	0 (0.0%)	0 (0.0%)	0 (0.0%)	
Days on ventilator	7	3.0 (1.8, 4.8)	3.5 (2.3, 4.8)	4.0 (4.0, 4.0)	0.96^1^
Days in ICU	7	4.0 (2.5, 5.3)	3.0 (2.0, 4.0)	4.0 (4.0, 4.0)	0.92^1^
Expired during hospital stay	500	4 (1.2%)	3 (2.6%)	1 (2.1%)	0.41^4^
Discharge destination: home	500	330 (98.2%)	112 (95.7%)	45 (95.7%)	0.17^4^
Hospital LOS (days)	500	1.0 (1.0-1.0)	1.0 (1.0-1.0)	1.0 (1.0- 2.0)	<0.001^1^
Adverse event					
No adverse event	500	283 (84.2%)	88 (75.2%)	37 (78.7%)	0.10^4^
Minor adverse event		8 (2.4%)	8 (6.8%)	1 (2.1%)	
Major adverse event		45 (13.4%)	21 (17.9%)	9 (19.1%)	

Clinical characteristics of patients who received PT or OT

Table 5 displays patient demographic and clinical characteristics by STS risk category for patients who received therapy. The results appear similar to what was seen in the entire cohort. Unlike those who did not receive therapy, there were significant differences in the rates of femoral vein TVT access site, in-hospital adverse events, and discharge home between groups. Similar to all patients, a higher percentage of patients in the acute rehabilitation low STS risk group were accessed via the femoral vein and discharged home compared to patients in the intermediate and high STS risk groups. Patients in the hospital-based rehabilitation low STS risk group had a lower percentage of adverse events compared to patients in the intermediate and high STS risk groups, similar to all patients.

**Table 5:** Summary of patient baseline characteristics, clinical, surgical, and therapy-related measures, and outcomes by STS risk category for patients who received therapy ^1^Kruskal-Wallis rank sum test; ^2^Pearson's chi-squared test; ^3^Fisher's exact test with simulated p-value; ^4^Fisher's exact test AM-PAC 6cBM - Activity Measure for Post-Acute Care "6 clicks" Basic Mobility score; CCI - Charlson Comorbidity Index; ICU - intensive care unit; JH-HLM - Johns-Hopkins Highest Level of Mobility score; KCCQ-12 - Kansas City Cardiomyopathy Questionnaire-12; LOS - length of stay; OT - occupational therapy; PT - physical therapy; STS - Society of Thoracic Surgeons; TVT - transcatheter valve therapy

Variable; median (IQR), n (%)	n	STS low risk, n=351	STS intermediate risk, n=273	STS high risk, n=197)	p-value
Age, years	821	76.0 (70.0-82.0)	81.0 (75.0-87.0)	83.0 (78.0-89.0)	<0.001^1^
Sex					
Male	821	193 (55.0%)	121 (44.3%)	62 (31.5%)	<0.001^2^
Female		158 (45.0%)	152 (55.7%)	135 (68.5%)	
Race					
American Indian/Alaska Native	821	2 (0.6%)	0 (0.0%)	0 (0.0%)	0.54^3^
Asian		3 (0.9%)	3 (1.1%)	6 (3.0%)	
Black		66 (18.8%)	46 (16.8%)	29 (14.7%)	
Hispanic		0 (0.0%)	0 (0.0%)	0 (0.0%)	
Native Hawaiian/Pacific Islander		0 (0.0%)	0 (0.0%)	1 (0.5%)	
White		255 (72.6%)	204 (74.7%)	143 (72.6%)	
Other		4 (1.1%)	3 (1.1%)	2 (1.0%)	
Do not know/declined		21 (6.0%)	17 (6.2%)	16 (8.1%)	
Race recategorized					
Black	821	66 (18.8%)	46 (16.8%)	29 (14.7%)	0.57^3^
White		255 (72.6%)	204 (74.7%)	143 (72.6%)	
Do not know/declined		21 (6.0%)	17 (6.2%)	16 (8.1%)	
Other		9 (2.6%)	6 (2.2%)	9 (4.6%)	
Insurance					
Medicaid	821	8 (2.3%)	5 (1.8%)	0 (0.0%)	<0.001^4^
Medicare		300 (85.5%)	243 (89.0%)	190 (96.4%)	
Private		43 (12.3%)	25 (9.2%)	7 (3.6%)	
Body mass index, kg/m^2^	817	29.5 (25.2-33.8)	28.1 (24.1-32.1)	26.9 (23.1-31.8)	<0.001^1^
CCI	821	3.0 (1.0-5.0)	4.0 (2.0-6.0)	5.0 (3.0-7.0)	<0.001^1^
Overall KCCQ-12	801	50.0 (31.8-72.5)	42.7 (24.1-61.5)	33.3 (17.5-53.9)	<0.001^1^
Number of previous cardiac surgeries	813	0.0 (0.0-0.0)	0.0 (0.0-1.0)	0.0 (0.0-1.0)	<0.001^1^
TVT access site					
Axillary artery	821	3 (0.9%)	5 (1.8%)	6 (3.0%)	0.03^3^
Direct aortic		0 (0.0%)	0 (0.0%)	0 (0.0%)	
Femoral artery		308 (87.7%)	237 (86.8%)	154 (78.2%)	
Subclavian artery		1 (0.3%)	5 (1.8%)	3 (1.5%)	
Transapical		1 (0.3%)	0 (0.0%)	0 (0.0%)	
Transcarotid		11 (3.1%)	7 (2.6%)	6 (3.0%)	
Transcaval		27 (7.7%)	17 (6.2%)	27 (13.7%)	
Transseptal via femoral vein		0 (0.0%)	2 (0.7%)	1 (0.5%)	
TVT access site					
Femoral artery	821	308 (87.7%)	237 (86.8%)	154 (78.2%)	0.007^4^
Subclavian artery		1 (0.3%)	5 (1.8%)	3 (1.5%)	
Other		42 (12.0%)	31 (11.4%)	40 (20.3%)	
TVT access site: femoral	821	308 (87.7%)	237 (86.8%)	154 (78.2%)	0.007^2^
Days on ventilator	48	5.0 (2.0-13.0)	3.0 (2.0-5.0)	2.0 (2.0-3.3)	0.22^1^
Days in ICU	142	2.0 (1.0-5.0)	3.0 (2.0-6.0)	3.0 (2.0-7.0)	0.37^1^
Expired during hospital stay	821	1 (0.3%)	5 (1.8%)	5 (2.5%)	0.04^4^
Average 5-meter walk score	414	6.3 (4.7-8.3)	7.0 (6.0-9.8)	8.5 (6.3-11.1)	<0.001^1^
Average 5-meter walk score 0-6 seconds	414	89 (45.6%)	40 (28.8%)	16 (20.0%)	<0.001^2^
Total rehab visits	821	2.0 (1.0-2.0)	2.0 (1.0-3.0)	2.0 (2.0-4.0)	<0.001^1^
Total OT visits	821	1.0 (0.0-1.0)	1.0 (1.0-1.0)	1.0 (1.0-2.0)	<0.001^1^
First JHHLM	800	7.0 (7.0-7.0)	7.0 (6.0-7.0)	7.0 (5.0-7.0)	<0.001^1^
Last JHHLM	800	7.0 (7.0-7.0)	7.0 (6.0-7.0)	7.0 (6.0-7.0)	<0.001^1^
First AM-PAC 6cBM	733	22.0 (18.0-24.0)	19.0 (17.0-23.0)	18.0 (17.0-20.0)	<0.001^1^
Last AM-PAC 6cBM	733	22.0 (18.0-24.0)	20.0 (17.8-24.0)	18.0 (17.0-21.0)	<0.001^1^
Hospital PT frequency (visits/hospital LOS)	821	0.6 (0.3-1.0)	0.5 (0.3-1.0)	0.5 (0.3-1.0)	0.001^1 ^
Hospital OT frequency (visits/hospital LOS)	821	0.5 (0.0-1.0)	0.5 (0.1-1.0)	0.3 (0.2-0.7)	0.36^1^
PT/OT post-procedure on day 1	785	290 (86.3%)	210 (82.0%)	140 (72.5%)	<0.001^2^
Functional status ambulatory	799	308 (89.8%)	215 (81.4%)	138 (71.9%)	<0.001^2^
Hospital LOS (days)	821	2.0 (1.0-3.0)	2.0 (1.0-4.0)	3.0 (1.0-7.0)	<0.001^1^
Discharge destination: home	821	325 (92.6%)	232 (85.0%)	149 (75.6%)	<0.001^2^
Adverse event					
None	821	259 (73.8%)	196 (71.8%)	126 (64.0%)	0.08^2^
Minor		13 (3.7%)	7 (2.6%)	12 (6.1%)	
Major		79 (22.5%)	70 (25.6%)	59 (29.9%)	

Patients who received PT or OT: timing of initiation of rehabilitation

Receipt of PT/OT on day 1 post-procedure was significantly associated with STS risk group (p<0.001). Day 1 post-procedure PT/OT receipt decreased as STS risk score group increased: 290/351 (86.3%), 210/273 (82.0%), and 140/197 (72.5%) likelihood for low, intermediate, and high STS risk groups, respectively (see Table 5). There was no significant difference in the odds of PT/OT receipt on day 1 between low and intermediate risk groups (OR: 0.72; 95% CI: 0.46-1.13; p=0.16). The odds of receiving PT or OT on day 1 post-procedure was 58% lower for the high-risk group when compared to the low-risk group (OR: 0.42; 95% CI: 0.27-0.65; p<0.001). Results were similar when adjusted for the occurrence of at least one adverse event, with 23% and 53% lower odds for intermediate and high risk groups, respectively (Table 3 and Figure 1).

Patients who received PT or OT: functional ambulation at discharge

Functional ambulation at discharge was significantly associated with the STS risk group. Patients were less likely to be functional ambulators as the STS risk score increased: 308/351 (89.8%), 215/273 (81.4%), and 138/197 (71.9%) for low, intermediate, and high STS risk groups, respectively (p<0.001) (Table 5). The odds of being functionally ambulatory for the intermediate group was 50% lower when compared to the low group (OR: 0.50; 95% CI: 0.31-0.80; p=0.004). Patients in the high STS risk group had 71% lower odds of being functionally ambulatory at discharge compared to the low STS risk group (OR: 0.29; 95% CI: 0.18-0.46; p<0.001). Results were nearly identical when adjusted for the occurrence of at least one adverse event at 50% and 70% (Table 3 and Figure 1).

## Discussion

The purpose of this study was to examine the relationship between the STS risk score and hospital-based PT or OT receipt, hospital LOS, and discharge rates to home versus not home for patients following TAVR. In patients who did receive therapy, the relationship of STS risk score and functional ambulation status at discharge and receipt of PT or OT on day 1 after the procedure was analyzed.

Low-risk patients had higher odds of being discharged home compared to other groups. LOS was longer for intermediate and high STS risk groups when compared to low STS risk groups. Patients in the high-risk STS group had higher odds of receiving PT or OT compared to other groups. Low-risk patients had higher odds of being functionally ambulatory at discharge. Patients in the low STS risk group were more likely to be evaluated on post-procedure day 1 compared to intermediate or high STS risk groups.

As expected, due to the nature of the STS risk scoring criteria, patients in the low-risk group were younger, more likely to be male, and had a lower comorbidity burden (Charlson Comorbidity Index scores of 2.0 for low, 4.0 for intermediate, and 5.0 for high risk groups; p<0.001). The observations of patients in intermediate and low STS risk groups being younger age correlate with other research in patients one year after TAVR [7]. The observed decrease in femoral access site usage as STS risk group increases is also expected, as arterial disease is considered in the STS risk group calculator, and research on access sites in TAVR acknowledges extensive coronary artery disease, vessel calcification, or anatomical differences as reasons that femoral access may not be utilized due to the increased risk for vascular complications [27].

The LOS being shorter for patients in the low STS risk group when compared to the intermediate and high-risk groups was anticipated. Based on the inputs used to calculate STS risk group, these patients tend to have fewer comorbidities, lower rates of adverse events, and more medical stability, allowing for expedited discharge compared to their high-risk peers. Additionally, patients in the low STS risk group were more likely to return home. A study by Zhoa et al. reported finding that a safe discharge destination accounted for 80% of non-medical related delays in discharge days, which can impact LOS for patients not returning home [28]. ​​​​​The increased odds of being functionally ambulatory at discharge for the patients in the low STS risk group are multifactorial. Patients in low STS risk groups may have better pre-surgery function, lower frailty scores (higher gait speed, higher Katz activities of daily living scores, and better nutrition markers such as albumin labs) compared to those in intermediate and high STS risk groups. Functional ambulation at discharge may play a role in a patient's need for placement, as those who are unable to ambulate at discharge may not be safe to return to their home. A single-center study of 145 patients following TAVR found that only 15% of patients reported they could walk easily, nearly half had no problems with self-care, and only 17% had no difficulties in performing daily activities on the Euro-Quality of Life [29]. Van der Wulp et al. found that 47.2% of individuals had impaired basic activities of daily living, while 36.1% had impaired instrumental activities of daily living, and 35.2% had impaired mobility measured via the Timed Up and Go test and gait speed [8]. Goel et al. found that optimizing a patient's physical function, including gait speed one year post-procedure, was correlated with better outcomes [30]. To address these findings, the patient education provided by rehabilitation therapists included post-discharge recommendations for stage II cardiac rehabilitation, a home walking program, and cardiac warning signs.

Despite higher odds of receiving therapy for patients in the high STS risk group, these patients were less likely to be seen on post-procedure day 1. The greater percentage of adverse events in the high STS risk group (33.2%) compared to the low STS risk group may indicate medical instability, which would prevent initiation of PT or OT evaluation on day 1 post-procedure. A lesser percentage of patients in the low STS risk group (21.1%) experienced an event, indicating more medical stability and greater opportunities to initiate therapy evaluation on post-procedure day 1.

Limitations

This study is subject to several limitations. These results should be interpreted with caution due to the single-site nature of this retrospective review. Due to the timeframe of this study, the potential impact of the COVID-19 pandemic on hospital policy and staffing, patient selection, LOS, and discharge location should be considered. The reasons why approximately 500 patients did not receive therapy remain unidentified, limiting the ability to fully interpret treatment allocation and introducing the potential for selection bias. Moreover, important frailty indicators, such as the Katz basic activities of daily living score and serum albumin levels, were not available, restricting our understanding of patient selection for hospital-based rehabilitation and the clinical decision-making process. Additionally, 75 patients experienced major clinical events, the majority of whom had been categorized as low risk. This discrepancy raises concerns regarding the sensitivity and accuracy of the risk stratification model employed. However, when adjusting ORs for the occurrence of at least one adverse event, results were nearly identical to unadjusted ORs. While over 95% of patients across all groups were ultimately discharged home, suggesting favorable short-term outcomes, this high discharge rate may mask important differences in recovery trajectories and post-discharge care needs. Future studies could distinguish patients who received PT or OT from those who did not. Rather than focusing solely on those who received therapy, future analyses should explore differences across the full spectrum of patients, both those who did and did not receive PT/OT, within each risk category. Such comparisons could help clarify the role of therapy across different functional risk profiles and inform more targeted rehabilitation strategies. 

## Conclusions

Patients with a higher STS risk score are more likely to receive PT or OT, to have longer LOS, and are less likely to return home. In the hospital-based rehabilitation group, patients with high-risk STS scores are less likely to be functionally ambulatory at discharge and less likely to be seen post-procedure on the first day. Development and implementation of a clinical pathway based on STS risk group may help with patient selection for hospital-based rehabilitation services.
